# The Risk of Fracture in Patients With Multiple Sclerosis: The UK General Practice Research Database

**DOI:** 10.1002/jbmr.418

**Published:** 2011-05-06

**Authors:** Marloes T Bazelier, Tjeerd van Staa, Bernard MJ Uitdehaag, Cyrus Cooper, Hubert GM Leufkens, Peter Vestergaard, Joan Bentzen, Frank de Vries

**Affiliations:** 1 Utrecht Institute of Pharmaceutical Sciences, Utrecht UniversityThe Netherlands; 2 General Practice Research Database, Medicines and Healthcare Products Regulatory AgencyLondon, UK; 3 MRC Lifecourse Epidemiology Unit, University of SouthamptonSouthampton, UK; 4 MS Center Amsterdam, VU University Medical CenterAmsterdam, The Netherlands; 5 Institute of Musculoskeletal Sciences, University of OxfordOxford, UK; 6 The Osteoporosis Clinic, Aarhus University HospitalAarhus, Denmark; 7 National Institute of Public Health, University of Southern DenmarkCopenhagen, Denmark; 8 Department of Clinical Pharmacy and Toxicology, Maastricht University Medical CentreMaastricht, The Netherlands

**Keywords:** MULTIPLE SCLEROSIS, FRACTURE RISK, OSTEOPOROSIS, EPIDEMIOLOGY

## Abstract

Patients with multiple sclerosis (MS) may be at an increased risk of fracture owing to a greater risk of falling and decreased bone mineral density when compared with the general population. This study was designed to estimate the relative and absolute risk of fracture in patients with MS. We conducted a population-based cohort study using data from the UK General Practice Research Database linked to the National Hospital Registry (1997–2008). Incident MS patients (*n* = 5565) were matched 1:6 by year of birth, sex, and practice with patients without MS (controls). Cox proportional-hazards models were used to derive adjusted hazard ratios (HRs) for fracture associated with MS. Time-dependent adjustments were made for age, comorbidity, and drug use. Absolute 5- and 10-year risks of fracture were estimated for MS patients as a function of age. Compared with controls, MS patients had an almost threefold increased risk of hip fracture [HR = 2.79, 95% confidence interval (CI) 1.83–4.26] and a risk of osteoporotic fracture that was increased 1.4-fold (HR = 1.35, 95% CI 1.13–1.62). Risk was greater in patients who had been prescribed oral/intravenous glucocorticoids (GCs; HR = 1.85, 95% CI 1.14–2.98) or antidepressants (HR = 1.79, 95% CI 1.37–2.35) in the previous 6 months. Absolute fracture risks were low in younger MS patients but became substantial when patients were older than 60 years of age. It is concluded that MS is associated with an increased risk of fracture. Fracture risk assessment may be indicated in patients with MS, especially those prescribed GCs or antidepressants. © 2011 American Society for Bone and Mineral Research

## Introduction

Multiple sclerosis (MS) is a neurodegenerative disease characterized by the gradual accumulation of focal plaques of demyelination, particularly in the periventricular areas of the brain. According to the World Health Organization, MS affects more than 1.3 million people worldwide, including 630,000 Europeans, 520,000 Americans, and 66,000 Eastern Mediterranean patients.(1)

Patients with MS may be at risk of fracture(2) owing to an increased risk of falling(3–5) and low bone mineral density (BMD).(6–10) An increased risk of falling may result from imbalance, disability, or spasticity.(3,5,11) Osteoporosis occurs more frequently among patients with MS, probably as a result of immobility,(7,10) a low level of daily activity,(6,12) vitamin D deficiency,(10) and the use of glucocorticoids (GCs).(6,7,13) Furthermore, it has been shown that patients with rheumatoid arthritis (RA), chronic obstructive pulmonary disease (COPD), or inflammatory bowel disease whose cumulative exposure to GCs was greater than 1 g (in prednisone equivalents) had a substantially increased risk of osteoporotic fracture.(14) MS patients who suffer from a relapse are often prescribed oral or intravenous methylprednisolone (MPH) with a dosage that corresponds to 1.5 to 3.3 g of prednisone equivalents. A recent study showed that in patients with RA, the underlying disease process also may contribute to the fracture risk.(15) In patients with MS, there may be a similar association between disease activity and risk of fracture: Inflammation, which is highest during relapses, may be associated with a reduction in BMD in MS patients.(8) However, in MS patients, individual risk profiles for fracture have not been determined.

The aim of this study was to determine the relative risk of fracture in patients with MS compared with population-based controls and to calculate the absolute 5- and 10-year risks of fracture in MS patients.

## Methods

### Data source

Information for this study was obtained from the General Practice Research Database (GPRD). The GPRD comprises prospectively collected computerized medical records for over 10 million patients under the care of general practitioners (GPs) in the United Kingdom (UK). It has been the source for numerous epidemiologic studies, and the accuracy and completeness of these data have been well validated and documented.(16) Previous studies of GPRD data have shown a high level of data validity with respect to the reporting of fractures.(17) GPRD data have been linked to the national Hospital Episode Statistics (HES) in England for approximately 45% of all practices.(18) In this study, data were linked from April 1997 until March 2008.

### Study population

The case population consisted of all patients aged 18 years or older with at least one recorded diagnosis of MS during the period of GPRD or HES data collection. For this study, GPRD data collection started in 1987 and ended in August 2009 and HES data collection started in April 1997 and ended in March 2008 (thereby defining the total study period as 1987 to 2009). Patients with a history of MS before the start of data collection were excluded, thereby restricting the case population to incident MS patients. Patients with MS were stratified to probable and possible cases at baseline (date of first diagnosis/index date). A *probable* case was defined as someone with a diagnosis of MS in the GPRD and a diagnosis of MS in the HES or a diagnosis of MS either in the GPRD or the HES and at least one of the following: (1) an MRI within 6 months of the first diagnosis, (2) two or more subsequent MS diagnoses (GPRD), and/or (3) a prescription for either corticotropin, glatiramer, interferon β1a or β1b, or natalizumab at any time during follow-up. All other patients with a diagnosis of MS in the GPRD or the HES were considered *possible* cases. The case definition of MS has been developed by a senior neurologist (BU) and a senior pharmacoepidemiologist who has 8 years' experience with analysis of GPRD (FdV). We defined patients free of osteoporosis treatment at baseline as those who had not been prescribed bisphosphonates, raloxifene, strontium ranelate, and/or parathyroid hormone (PTH) before the start of follow-up.

Each MS patient was matched by year of birth, sex, and practice to six control individuals (patients without a diagnosis of MS at any time during their period of registration). The index date of MS diagnosis was the date of the first record of MS after the start of valid data collection. Control patients were assigned the same index date as their matched case. Each patient then was followed from his or her index date to the end of data collection, the date of transfer of the patient out of the practice area, or the patient's death, whichever came first.

### Study outcomes

All patients (cases and controls) were followed up for the occurrence of fractures. The types of fracture were classified according to the International Classification of Diseases (ICD-10) categories. These included skull (S02), neck (S12), ribs (S22), pelvis (S32), shoulder (S42), forearm (S52), hand (S62), hip/femur (S72), ankle (S82), foot (S92), or unspecified fractures (T02, T08, T10, T12). A clinical osteoporotic fracture was defined as a fracture of the radius/ulna, vertebrae, femur, hip, humerus, pelvis, or ribs.

The total follow-up period was divided into 30-day intervals. The presence of risk factors was assessed by reviewing the computerized medical records for any evidence of risk factors before the start of an interval. Potential confounders that were determined at baseline included sex, body mass index (BMI), smoking status, alcohol use, history of falling, and history of fracture. Potential confounders that were determined for a time-dependent analysis included age, a history of chronic diseases (congestive heart failure, RA, cerebrovascular disease,(19) inflammatory bowel disease, dementia, depression, epilepsy, and COPD(20)), as well as evidence of fatigue or visual disturbances 6 months before the start of a 30-day interval. A prescription for orally or intravenously administered GCs,(14,21) statins,(22) antiarrythmics, antidiabetics, antidepressants,(23) antipsychotics,(24) hypnotics/anxiolytics, asthma medication, anticonvulsants, hormone-replacement therapy, vitamin D, levothyroxine, baclofen, or opioids (potencies equivalent to tramadol or higher) in the previous 6 months also were considered as potential confounders. The prescription of a medication was used as a proxy for use of that drug.

In the United Kingdom, exacerbations of MS are treated with a short course of orally or intravenously administered MPH.(25) Prescriptions can be issued by the GP, or the patient may be referred to an MS clinic for intravenous MPH on 3 consecutive days. Information on MPH exposure during MS relapses was retrieved from anonymous free text, as recorded by the GP or by the consultant neurologist, in a discharge summary or letter to the GP. We searched all free-text records of each MS patient with the following search terms: *methylpredniso*, *methyl-predniso*, *methyl predniso*, *solumedrol*, *solu medrol*, *solu-medrol*, *ivmp*, *medrone*, *ivmp*, *booster*, *bolus*, *pulse ther*, and *steroid pulse*. Records then were made anonymous, and the date of administration, MPH dose, and route of administration were noted. Average daily dose and cumulative dose of oral/intravenous GCs were determined for the 6-month period before the start of an interval. The average daily dose was defined as the total dose of GCs (in prednisolone equivalents) that was prescribed in the previous 6 months divided by the number of days.

In the analysis to derive absolute risk, additional specific risk factors were considered, including vertigo, dizziness, imbalance, disturbance of sensation, spasticity, sexual dysfunction, paroxysmal symptoms, cognitive dysfunction, vitamin D deficiency, and proxy indicators of increased disability (i.e., home visits by the GP, nursing care, and patient receiving residential care/living in a care home or using a wheelchair or walking aid) 6 months before the start of an interval. Furthermore, the use of nonsteroidal anti-inflammatory drugs (NSAIDs), meprobamate, tizanidine, dantrolene, modafinil, methylphenidate, or amantadine in the previous 6 months was included in this analysis.

To create a cohort of patients unexposed to osteoporosis treatment at baseline, medication records of all participants of the study (MS cases and controls) were searched for prescriptions of bisphosphonates, raloxifene, strontium ranelate, and PTH.

### Statistical analysis

We compared baseline characteristics between cases and controls using chi-square testing. Two main analyses were conducted using Cox proportional-hazards models. The first analysis compared the fracture risk in MS patients with that in control patients to yield an estimate of the relative risk [as a hazard ratio (HR)] of fracture in MS. In that analysis, the calculations were adjusted for all potential confounders that changed the HR more than 1% in an age/sex-adjusted analysis. Analyses were stratified to duration of disease, drug use in the previous 6 months (including daily and cumulative exposure to GCs), falling 3 months to 1 year before, and a history of fatigue and the disability proxy indicator in the previous 6 months. In addition to the full cohort analysis, we calculated HRs in an osteoporosis-treatment-naive cohort, where we restricted the analysis to MS patients and controls free of osteoporosis treatment at baseline.

In the second analysis, we calculated the long-term risk of osteoporotic and hip fracture using the full cohort. The Cox model allows calculation of an individual's probability of fracture (i.e., survivor function) for each set of patient characteristics. For the analysis of long-term risk, we fitted the regression model with the general and specific risk factors, which were determined at baseline, using forward selection. All characteristics, except age, were included as categorical variables in the regression models. For the variables of age, sex, and each of the risk factors, we also investigated possible statistical interactions with MS, although none was added subsequently to the model. The 5- and 10-year risks of osteoporotic and hip fracture then were estimated, conditional on patient survival. We evaluated risks for MS patients in different sex and age categories using their individual risk profiles. In addition, we estimated risks with GC or antidepressant use, adding the corresponding risk factor to the model. Various methods were used to test the fitting of the Cox models, including a test of the proportional-hazards assumption. We also compared the observed 5-year probability of fracture (based on the Kaplan-Meier estimate) with the probability predicted by the Cox model. To assess the internal validity of the model further, the *C*-statistic was calculated, and we performed a 10-fold cross-validation. We applied the shrinkage factor that we found to the β coefficients of the model, and we adjusted the *C*-statistic for overestimation.

A sensitivity analysis was conducted to assess the impact of defining the index date as the first MS diagnosis 1 year after start of follow-up. In another sensitivity analysis, we excluded all probable MS patients who were classified as probable based on their MRI. All data management and statistical analyses were conducted using SAS Version 9.1/9.2 software (SAS Institute, Inc., Cary, NC, USA).

## Results

The study population included 5565 patients with MS and 33,360 population-based controls. The mean age at index date (first diagnosis of MS) was 44.8 years, and 70.0% of the MS patients were female. The mean duration of follow-up after the index date was 5.7 years for the MS patients and 6.0 years for the control individuals. Before the index date, 32.2% of the MS patients had been prescribed at least one antidepressant, 10.0% at least one anticonvulsant, and 13.2% at least one oral/intravenous GC. In control patients, these proportions were 21.2%, 2.5%, and 6.4%, respectively. Fractures were recorded during follow-up in 394 MS patients (7.1%) and 1742 control individuals (5.2%). Of all MS patients, 3386 were classified as probable and 2179 as possible cases at baseline. Further descriptive details of the participants are given in Table 1.

**Table 1:** Baseline Characteristics of MS Patients and Controls

Characteristic	Cases (%) (*n* = 5565)		Controls (%) (*n* = 33,360)	
Female	3897	(70.0)	23,365	(70.0)
Age at index date				
Mean	44.8		44.7	
By category				
18–29	670	(12.0)	4018	(12.0)
30–39	1444	(25.9)	8663	(26.0)
40–49	1617	(29.1)	9700	(29.1)
50–59	1066	(19.2)	6391	(19.2)
60+	768	(13.8)	4588	(13.8)
Mean duration of disease (years) [95% CI]	5.7 [5.5–5.8]		6.0 [6.0–6.1]	
Smoking				
Never	2135	(38.4)	15,177	(45.5)^a^
Current	1543	(27.7)	7173	(21.5)^a^
Ex-smoker	815	(14.6)	4287	(12.9)^a^
Unknown	1072	(19.3)	6723	(20.2)
BMI				
<20	443	(8.0)	1957	(5.9)^a^
20–25	1926	(34.6)	11,384	(34.1)
25–30	1318	(23.7)	8803	(26.4)^a^
>30	888	(16.0)	5476	(16.4)
Unknown	990	(17.8)	5740	(17.2)
Disease history				
Fracture	829	(14.9)	4498	(13.5)^a^
Falling	362	(6.5)	995	(3.0)^a^
Fatigue	441	(7.9)	1752	(5.3)^a^
Asthma	582	(10.5)	3478	(10.4)
COPD	56	(1.0)	310	(0.9)
Congestive heart failure	33	(0.6)	157	(0.5)
Diabetes mellitus	157	(2.8)	854	(2.6)
Rheumatoid arthritis	34	(0.6)	234	(0.7)
Cerebrovascular incident	158	(2.8)	390	(1.2)^a^
Epilepsy	132	(2.4)	448	(1.3)^a^
History of drug use				
Statins	277	(5.0)	1278	(3.8)^a^
Antiarrythmics	32	(0.6)	163	(0.5)
Antidiabetics	130	(2.3)	684	(2.1)
Antidepressants	1794	(32.2)	7066	(21.2)^a^
Antipsychotics	226	(4.1)	953	(2.9)^a^
Anxiolytics/hypnotics	1187	(21.3)	5048	(15.1)^a^
Anticonvulsants	558	(10.0)	823	(2.5)^a^
Opioids	386	(6.9)	1217	(3.6)^a^
Oral/intravenous glucocorticoids	737	(13.2)	2132	(6.4)^a^

CI = confidence interval; BMI = body mass index; COPD = chronic obstructive pulmonary disease.

a Statistically significant difference (*p* < .05) between cases and controls based on chi-square test.

Table 2 shows that patients with MS had a 1.2-fold increased risk of any fracture: adjusted HR = 1.23 [95% confidence interval (CI) 1.09–1.38]. The risk of osteoporotic fracture was increased 1.4-fold (HR = 1.35, 95% CI 1.13–1.62), and the risk of hip fracture was increased almost 3-fold (HR = 2.79, 95% CI 1.83–4.26). The risk of vertebral or radius/ulna fracture was not increased. When we compared probable MS patients with possible MS patients, we found that the risk of osteoporotic fracture was higher in probable patients: HR = 1.46 (95% CI 1.19–1.79) in probable and HR = 1.14 (95% CI 0.84–1.54) in possible MS patients, although the difference was not significant (*p* = .152). There was a significant different risk of hip fracture between probable MS patients (HR = 3.75, 95% CI 2.32–6.07) and possible MS patients (HR = 1.64, 95% CI 0.81–3.32). Because the risks of fracture in probable patients were comparable with the risks in all MS patients, we performed the subsequent analyses in the full MS cohort, thereby increasing the number of patients. The risks in the osteoporosis-treatment-naive cohort (5494 MS patients, 32,669 controls) were similar to those in all MS patients.

**Table 2:** Risk of Fracture in MS Patients Compared With Control Patients, by Type of Fracture

	Full cohort analysis				Osteoporosis-treatment-naïve analysis			
		
	Cases (*n*a = 5565); Controls (*n* = 33,360)				Cases (*n* = 5494); Controls (*n* = 32,669)			
		
	Fracture, *n*	Rate/1000 person-years	Age-sex-adjusted HR (95% CI)	Fully adjusted HR (95% CI)	Fracture, *n*	Rate/1000 person-years	Age-sex-adjusted HR (95% CI)	Fully adjusted HR (95% CI)
No MS	1742	8.6	1	1	1686	8.5	1	1
MS								
Any fracture	394	12.5	1.52 (1.36–1.69)	1.23 (1.09–1.38)^a^	381	12.2	1.50 (1.34–1.68)	1.22 (1.09–1.38)^a^
Osteoporotic^j^	173	5.5	1.73 (1.46–2.04)	1.35 (1.13–1.62)^b^	163	5.2	1.68 (1.41–1.99)	1.31 (1.09–1.57)^b^
Hip	37	1.2	3.83 (2.58–5.67)	2.79 (1.83–4.26)^c^	36	1.2	4.20 (2.80–6.31)	3.05 (1.97–4.73)^g^
Vertebral	8	0.3	1.21 (0.57–2.57)	0.94 (0.43–2.02)^d^	7	0.2	1.19 (0.53–2.65)	0.93 (0.41–2.13)^h^
Radius/ulna	65	2.1	1.43 (1.10–1.88)	1.16 (0.87–1.55)^e^	61	2.0	1.37 (1.04–1.81)	1.12 (0.83–1.50)^e^
Other	67	2.1	1.65 (1.26–2.16)	1.27 (0.95–1.69)^f^	62	2.0	1.55 (1.17–2.04)	1.19 (0.89–1.60)^f^

HR = hazard ratio; CI = confidence interval.

a Adjusted for (i) and the use of opioids in the previous 6 months, history of cerebrovascular disease, epilepsy.

b Adjusted for (i) and the use of opioids in the previous 6 months, history of cerebrovascular disease, epilepsy, BMI.

c Adjusted for (i) and the use of opioids in the previous 6 months, history of fatigue in the previous 6 months, BMI.

d Adjusted for age, sex, the use of oral/intravenous glucocorticoids, antidepressants, opioids in the previous 6 months, history of fracture at index date.

e Adjusted for (i) and history of epilepsy, history of visual disturbance in the previous 6 months.

f Adjusted for (i) and the use of opioids in the previous 6 months, history of cerebrovascular disease, epilepsy.

g Adjusted for age, sex, the use of oral/intravenous GCs, antidepressants, anticonvulsants, opioids in the previous 6 months, history of falling at index date, history of fracture at index date, history of smoking, BMI.

h Adjusted for age, sex, the use of oral/intravenous glucocorticoids, antidepressants in the previous 6 months, history of fracture at index date.

^i^Age, sex, the use of oral/intravenous glucocorticoids, antidepressants, hypnotics/anxiolytics, anticonvulsants in the previous 6 months, history of falling at index date, history of fracture at index date, history of smoking.

l The numbers in the subcategories of osteoporotic fracture do not add up precisely because a patient can sustain more than one fracture, and therefore different types of fractures, on the same date.

Table 3 shows that the risk of osteoporotic fracture with any GC use in the previous 6 months was increased (HR = 1.85, 95% CI 1.14–2.98). The risk was doubled in patients who had recently been exposed to daily dosages of 7.5 mg of prednisolone equivalents or more compared with control patients (HR = 2.35, 95% CI 1.35–4.12). In addition, we found a similar increase in risk of osteoporotic fracture when we evaluated exposure to cumulative doses of 1 g or more of prednisolone equivalents in the previous year (HR = 2.35, 95% CI 1.34-4.10; data not shown). The fracture risk with recent GC use was higher in patients who had been prescribed a short course in the previous year (HR = 2.67, 95% CI 1.26–5.68), the risk being greater with orally administered GCs (HR = 4.65, 95% CI 1.88–11.51) than with intravenously administered GCs (HR = 1.39, 95% CI 0.44–4.45; data not shown). In patients who had been treated recently with antidepressants, the risk of osteoporotic fracture was almost doubled. This also was apparent for MS patients with a record of disability in the previous 6 months. For patients with a record of falling 1 year to 3 months earlier, the HR was 2.23 (95% CI 1.10–4.52). No association between the duration of disease (from the first MS diagnosis) and risk of fracture was apparent. In the osteoporosis-treatment-naive analysis, we found similar risks as in the full cohort analysis.

**Table 3:** Risk of Osteoporotic Fracture in MS Patients Compared With All Control Patients, by History of Drug Use and Disease Indicators

	Full cohort analysis				Osteoporosis-treatment-naive analysis			
		
	Cases (*n* = 5565); Controls (*n* = 33,360)				Cases (*n* = 5494); Controls (*n* = 32,669)			
		
	Fracture, *n*	%	Fully adjusted HR (95% CI)^a^		Fracture, *n*	%	Fully adjusted HR (95% CI)^a^	
No MS	698	2.1	1		668	2.0	1	
MS								
Osteoporotic fracture	173	3.1	1.35	(1.13–1.62)	163	3.0	1.31	(1.09–1.57)
Duration of disease								
<1 year	26	15.0	1.28	(0.86–1.91)	22	13.5	1.13	(0.73–1.73)
1–5 years	82	47.4	1.45	(1.14–1.84)	77	47.2	1.39	(1.09–1.78)
>5 years	65	37.6	1.28	(0.98–1.67)	64	39.3	1.29	(0.99–1.68)
History of drug use in previous 6 months								
Antidepressants								
Yes	67	38.7	1.79	(1.37–2.35)^b^	59	36.2	1.61	(1.21–2.15)
No	106	61.3	1.28	(1.04–1.58)	104	63.8	1.28	(1.04–1.58)
Antipsychotics								
Yes	5	2.9	1.75	(0.72–4.28)	4	2.5	1.48	(0.55–4.01)
No	168	97.1	1.35	(1.13–1.61)	159	97.5	1.31	(1.09–1.57)
Hypnotics/anxiolytics								
Yes	29	16.8	1.48	(1.00–2.19)	27	16.6	1.47	(0.98–2.20)
No	144	83.2	1.34	(1.11–1.62)	136	83.4	1.29	(1.07–1.57)
Anticonvulsants								
Yes	38	22.0	1.69	(1.20–2.39)	35	21.5	1.65	(1.15–2.36)
No	135	78.0	1.33	(1.10–1.61)	128	78.5	1.30	(1.07–1.57)
Oral/intravenous glucocorticoids								
No use	155	89.6	1.34	(1.11–1.61)	148	90.8	1.31	(1.09–1.58)
Any use	18	10.4	1.85	(1.14–2.98)	15	9.2	1.64	(0.98–2.77)
By average daily dose (mg prednisolone equivalents)								
<7.5 mg	5	2.9	1.19	(0.49–2.87)	5	3.1	1.30	(0.54–3.15)
≥7.5 mg	13	7.5	2.35	(1.35–4.12)	10	6.1	1.89	(1.01–3.57)
History of falling (3 months to 1 year before)								
Yes	8	4.6	2.23	(1.10–4.52)	7	4.3	2.05	(0.96–4.35)
No	165	95.4	1.36	(1.13–1.63)	156	95.7	1.32	(1.09–1.58)
History of MS indicators 6 months before								
Fatigue								
Yes	5	2.9	2.35	(0.97–5.70)	5	3.1	2.41	(0.99–5.86)
No	168	97.1	1.34	(1.12–1.60)	158	96.9	1.29	(1.07–1.56)
Disability proxy								
Yes	24	13.9	1.82	(1.19–2.78)	19	11.7	1.53	(0.96–2.45)
No	149	86.1	1.31	(1.08–1.58)	144	88.3	1.29	(1.07–1.56)

HR = hazard ratio; CI = confidence interval.

a Adjusted for age, sex, the use of oral/intravenous glucocorticoids, antidepressants, hypnotics/anxiolytics, anticonvulsants, opioids in the previous 6 months, history of falling at index date, history of fracture at index date, history of cerebrovascular disease, epilepsy, history of smoking, BMI.

b Statistically significant difference (*p* < .05) between MS patients with a history of medication use and MS patients unexposed to the same class of medication based on Wald test.

**Fig. 1:**
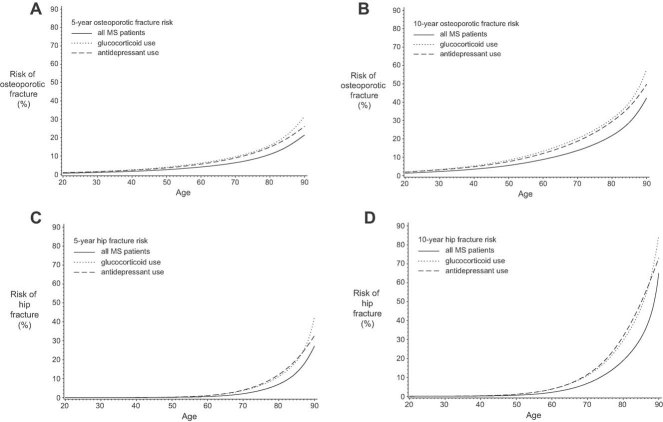
Five- and 10-year risks of osteoporotic and hip fractures (%) in MS patients in relation to age [306 × 197 mm (600 × 600 dpi)].

Figure 1 displays the 5- and 10-year risks of osteoporotic and hip fractures (percentages) in MS patients as a function of their age. In addition, separate curves were added for the use of oral/intravenous GCs or antidepressants in the previous 6 months. The distribution of the 5-year fracture risks in MS patients by sex and age categories is shown in Table 4. For example, among women aged 70 to 79 years, the median 5-year risk of osteoporotic fracture was 9.0%. However, there was considerable variation in the risk of osteoporotic fracture in this age range because the risk was 6.2% for the women in the 5th percentile of the risk profile and 22.3% for the women in the 95th percentile of the risk profile. The *C*-statistic was moderate (0.69) for the prediction of osteoporotic fracture and excellent (0.89) for the prediction of hip fracture.

**Table 4:** Five-Year Risk of Fracture in MS Patients at the 5th, 50th, and 95th Percentiles of Risk Profiles

	Osteoporotic fracture			Hip fracture		
		
	5th	50th	95th	5th	50th	95th
Women						
18–49	0.7	1.6	3.8	0.0	0.1	0.3
50–59	2.2	3.4	8.2	0.1	0.3	1.3
60–69	3.4	5.7	13.0	0.4	1.1	3.7
70–79	6.2	9.0	22.3	1.3	3.8	15.3
80+	9.8	15.8	35.2	6.4	12.1	46.0
Men						
18–49	0.5	1.2	3.0	0.0	0.1	0.3
50–59	1.6	2.4	5.7	0.1	0.3	1.1
60–69	2.5	3.7	8.5	0.4	1.0	3.0
70–79	3.7	5.7	18.9	1.1	2.8	12.0
80+	6.8	12.7	25.5	5.8	8.7	53.5

Compared with our primary analysis (Table 2), a sensitivity analysis yielded similar results when we used a lead-in time of 1 year after the start of data collection. Defining the index date as the first MS diagnosis after 1 year of data collection reduced the number of MS patients to *n* = 4339. The fully adjusted HR for any fracture was 1.22 (95% CI 1.07–1.39); for osteoporotic fracture, it was 1.40 (95% CI 1.15–1.70); and for hip fracture, the HR was 2.75 (95% CI 1.71–4.41). Exclusion of all probable MS patients who were classified as probable based on their MRI resulted in exclusion of 130 patients from the analysis, and the HRs for osteoporotic and hip fractures did not change substantially: HR = 1.45 (95% CI 1.18–1.79) for osteoporotic fracture and HR = 3.72 (95% CI 2.29–6.05) for hip fracture.

## Discussion

In this study we found that for patients with MS, the risk of hip fracture was almost three times higher than for control patients, and the risk of osteoporotic fracture was increased 1.4-fold. In MS patients who had been treated recently with oral or intravenous GCs or antidepressants, the risk of osteoporotic fracture was almost doubled compared with control individuals. Absolute fracture risks were low in younger MS patients but became substantial when patients were older than 60 years of age.

Our findings are in line with those of other studies that have compared BMD in patients with MS with that in healthy controls,(6–10) and they support the hypothesis that MS is associated with an elevated risk of fracture.(2) Cosman and colleagues performed a prospective cohort study on BMD in MS patients and controls. At baseline, the authors examined the history of fractures and found significantly more fractures in MS patients than in controls. In addition, BMD in patients with MS was lower than in age-matched control individuals—almost 1 SD lower in the spine and 1 to 1.6 SD lower in the femoral neck.(6) Formica and colleagues found that total-body bone mineral content was decreased in patients with MS (*Z*-score −0.3 ± 0.1 SD).(7) Weinstock-Guttman and colleagues reported that 80% of the male MS patients in their study had a reduced BMD at either the lumbar spine or the femoral neck. More specifically, 43% had osteopenia (−2.5 < *T*-score ≤ −1), and 37% had osteoporosis (*T*-score ≤ −2.5).(8) Among women with MS, 38% had osteopenia and 44% had osteoporosis. Ozgocmen and colleagues(10) found that BMD of the lumbar spine was nearly 1 SD lower in MS patients than in the healthy reference population.

There are various explanations for the increased risk of hip fracture in patients with MS. The etiology may be related to falling or decreased BMD. It has been shown that patients with MS have an increased risk of falling compared with healthy patients.(3–5) Symptoms of MS include muscle weakness, balance problems, uncoordinated movements, stiffness, numbness, tingling, blurred vision, fatigue, and dizziness.(26) Each symptom could play a role in the etiology of falling. Previous studies have investigated the role of disability as a risk factor for falling in patients with MS. Nilsagård and colleagues(3) reported the occurrence of falls in MS patients with an Expanded Disability Status Score (EDSS) between 3.5 and 6.0. They found that the odds of falling were doubled for each whole step on the EDSS. Finlayson and colleagues(5) evaluated self-reported falls in 1089 patients with MS and found that never or occasional use of a wheelchair approximately doubled the risk of a fall compared with the use of a wheelchair all the time. These results suggest that, on the one hand, disability may increase the risk of falling and, on the other, that if a patient always uses a wheelchair, that actually may protect against falling. However, patients who use a wheelchair have decreased mobility and consequently a reduced BMD and ultimately increased risk of fracture. Therefore, the association between disability and risk of fracture is ambiguous. In our study, we found that for patients with a record of falling 1 year to 3 months before or with a record of our proxy indicator of disability in the previous 6 months, the risk of osteoporotic fracture was approximately doubled.

A reduced BMD in MS may be caused by the patient's immobility,(7,10) vitamin D deficiency,(10) or use of GCs(6,7,13) or antidepressants.(23) Bone loss associated with physical inactivity can be explained by increased renal calcium losses, decreased gastrointestinal calcium absorption, secondary hyperparathyroidism, and increased bone turnover with depression of bone formation.(7) It has been demonstrated that patients with MS have lower levels of vitamin D than the general population,(10) which also may contribute to a reduced BMD in MS. Furthermore, MS is an inflammatory autoimmune disorder.(27) The inflammation, which is highest during relapses, may be associated with bone loss in MS patients.(8)

GC-induced osteoporosis involves systemic effects or direct effects on bone cells leading to induction of apoptosis in osteoblasts and osteocytes or suppression of their differentiation.(28,29) In this study we found that patients who were prescribed oral or intravenous GCs had higher fracture risks and that this relationship was dose-dependent. In patients who had been prescribed 7.5 mg or more of prednisone equivalents per day in the previous 6 months, the risk of osteoporotic fracture was almost doubled compared with control individuals. This result is in line with earlier studies that have linked GC use with increased fracture risk.(13,20,30,31) During relapses, patients with MS are often prescribed high doses of MPH. We found that in patients who had been prescribed short courses recently, the risk of fracture was increased further. However, we cannot exclude that the increased risk is due in part to an increased physical impairment caused by a more active disease resulting in GC treatment. Another explanation for the increased risk of fracture might be the greater use of antidepressants in MS patients. The use of antidepressants has been associated with a fracture risk that is approximately double that of control individuals.(32) This may be caused by use of the antidepressant itself, which could affect the microarchitecture of bone(33–35) or could lead to falls.(36) The underlying disease also may play a role in the increased fracture risk.(37)

Our study has many strengths. As far as we know, we are the first to estimate the risk of fracture in patients with MS compared with healthy control individuals using a large population-based cohort and the first to estimate absolute fracture risks in MS patients. Our source population was representative of the total UK population, and we had detailed longitudinal information on drug prescribing and other risk factors for fracture, such as low BMI and smoking status. The link with HES data allowed us to validate the diagnosis of MS in two independent disease registries.

Our study has some limitations, however. The first symptoms of MS can arise several years before a patient is diagnosed with MS, and therefore, the date of diagnosis as recorded on the GPRD is not entirely reliable. The mean age at index date (first diagnosis) was 44 years in our study, which is older than the typical age of MS onset.(38) However, since the association between duration of disease (from the first MS diagnosis) and risk of fracture was very weak, we believe that this would not change our results substantially. We conducted a sensitivity analysis using a lead-in period of 1 year after the start of data collection and found similar results as in our original analysis. The reliability of the order of sequence of an MRI and GPRD/HES diagnosis has not been validated. However, exclusion of probable MS patients who were classified as probable based on their MRI did not substantially change our findings. We did not have routinely collected information on the degree of disability in MS patients or on the course of their disease (i.e., relapsing-remitting or primary or secondary progressive). Because our source population was representative of the total UK population and there is no reason to believe that differential recording of MS would exist according to its classification, we may assume that approximately 85% to 90% of our MS patients had relapsing-remitting MS.(39) Although we constructed a proxy indicator for disability, we have not been able to account for all confounding. Furthermore, as already mentioned, the increased risk of fracture that we found with the exposure to short courses of GCs may be caused by either the GCs itself or worsening of MS during these relapses. Data on methylprednisolone use that has been prescribed during exacerbations may not have been complete because they were obtained from free-text fields written by GPs and discharge letters from neurologists. Based on a large clinical trial in patients with MS,(40) the average annualized relapse rate in patients with MS from the general population was 0.33; we have recorded 2181 treated relapses within 31,498 person-years, which implies a potential underrecording rate of 79.0%. This may have led to a nondifferential misclassification and an underestimation of the 2.7-fold increased risk of osteoporotic fracture in patients who had been prescribed a short course of methylprednisolone in the previous year. The GPRD does not routinely collect vitamin D levels or BMD measurements. The numbers of vertebral and rib fractures recorded in this study probably are underreported, which could have lead to a nondifferential misclassification. The true associations between MS and risk of any fracture/osteoporotic fracture therefore may be greater than reported in our study. We found no association between MS and vertebral fracture, but a true association may have been masked. The prescription of drugs had to be used as proxy for exposure because we could not confirm compliance nor account for the use of medications available over the counter in the United Kingdom.

In conclusion, we found that patients with MS had an increased risk of osteoporotic fracture and especially hip fracture. The risk was higher in patients who had recently used oral/intravenous GCs or antidepressants. Fracture risk assessment may be indicated in MS patients in particular when they have recently been prescribed antidepressants or high doses of GCs.

## Disclosures

The Department of Pharmacoepidemiology and Clinical Pharmacology, Utrecht Institute of Pharmaceutical Sciences, employing authors Marloes Bazelier, Tjeerd-Pieter van Staa, Hubert Leufkens, and Frank de Vries, has received unrestricted funding for pharmacoepidemiological research from GlaxoSmithKline, Novo Nordisk, the private-public funded Top Institute Pharma (http://www.tipharma.nl, includes co-funding from universities, government, and industry), the Dutch Medicines Evaluation Board, and the Dutch Ministry of Health. Tjeerd van Staa also works for the General Practice Research Database (GPRD), UK. The GPRD is owned by the UK Department of Health and operates within the Medicines and Healthcare products Regulatory Agency (MHRA). GPRD is funded by the MHRA, Medical Research Council, various universities, contract research organizations and pharmaceutical companies. Bernard Uitdehaag has received honoraria for consultancy from Novartis, Merck Serono, and Synthon.
